# IgA Nephropathy: Pleiotropic impact of Epstein-Barr virus infection on immunopathogenesis and racial incidence of the disease

**DOI:** 10.3389/fimmu.2023.1085922

**Published:** 2023-02-07

**Authors:** Jiri Mestecky, Bruce A. Julian, Milan Raska

**Affiliations:** ^1^ Department of Microbiology, University of Alabama at Birmingham, Birmingham, AL, United States; ^2^ Laboratory of Cellular and Molecular Immunology Institute of Microbiology, Czech Academy of Sciences, Prague, Czechia; ^3^ Department of Medicine, University of Alabama at Birmingham, Birmingham, AL, United States; ^4^ Department of Immunology, Faculty of Medicine and Dentistry, Palacky University and University Hospital, Olomouc, Czechia

**Keywords:** IgA nephropathy, Epstein-Barr virus, galactose-deficient IgA1, IgA system maturation, age of infection, virus spread

## Abstract

IgA nephropathy (IgAN) is an autoimmune disease in which poorly galactosylated IgA1 is the antigen recognized by naturally occurring anti-glycan antibodies, leading to formation of nephritogenic circulating immune complexes. Incidence of IgAN displays geographical and racial disparity: common in Europe, North America, Australia, and east Asia, uncommon in African Americans, many Asian and South American countries, Australian Aborigines, and rare in central Africa. In analyses of sera and cells from White IgAN patients, healthy controls, and African Americans, IgAN patients exhibited substantial enrichment for IgA-expressing B cells infected with Epstein-Barr virus (EBV), leading to enhanced production of poorly galactosylated IgA1. Disparities in incidence of IgAN may reflect a previously disregarded difference in the maturation of the IgA system as related to the timing of EBV infection. Compared with populations with higher incidences of IgAN, African Americans, African Blacks, and Australian Aborigines are more frequently infected with EBV during the first 1-2 years of life at the time of naturally occurring IgA deficiency when IgA cells are less numerous than in late childhood or adolescence. Therefore, in very young children EBV enters “non-IgA” cells. Ensuing immune responses prevent infection of IgA B cells during later exposure to EBV at older ages. Our data implicate EBV-infected cells as the source of poorly galactosylated IgA1 in circulating immune complexes and glomerular deposits in patients with IgAN. Thus, temporal differences in EBV primo-infection as related to naturally delayed maturation of the IgA system may contribute to geographic and racial variations in incidence of IgAN.

## 1 Introduction

IgA nephropathy (IgAN) is an autoimmune disease in which IgA, exclusively of the IgA1 subclass, with an altered glycan moiety manifested as deficiency of galactose (Gd-IgA1) on its heavy-chain hinge region (HR) acts as an autoantigen which is recognized by ubiquitous, naturally occurring, anti-glycan antibodies to form nephritogenic circulating immune complexes (CIC). Some of these complexes deposit in the glomerular mesangium to induce kidney injury (1–7). Detailed analyses of CIC and the mesangial immune deposits of IgAN patients revealed the presence of under-galactosylated IgA1 in the polymeric (p) form with joining (J) chain (3, 8–22). The nephritogenic potential of CIC containing Gd-IgA1 isolated from IgAN patients was demonstrated by studies of their effect on mesangial cells (23–25) or that of complexes generated *in vitro* with poorly galactosylated myeloma pIgA1 and corresponding anti-glycan antibodies (26) on the proliferation and activation of human primary mesangial cells in culture. Importantly, complexes with molecular mass of ~700-1,000 kDa displayed stimulatory activity whereas smaller complexes did not (3, 25). Furthermore, intravenous injection of complexes comprised of human Gd-pIgA1 and recombinant IgG antibodies specific for this IgA1 into immune-deficient mice induced kidney pathology features and urinary abnormalities typical of IgAN (27).

IgAN is the leading cause of primary glomerulonephritis in many countries (1). However, its prevalence displays striking geographic, racial, and age-related distributions (1, 28–30). IgAN is common in most European countries, USA, and east Asia but is less frequent in South America, India, Bangladesh, Indonesia, Nepal, Pakistan, and other Asian countries and is rare in central Africa (29–31). Furthermore, there are marked racial differences in disease incidence. African Blacks, African Americans, and indigenous Australian Aborigines living in remote rural areas display a low incidence of IgAN (31–41). Because of these race-associated differences in the incidence of the disease and discovery of multiplex families with multiple affected members, it has been proposed that genetically modulated differences play an important role in the mechanisms of disease (42–48).

Investigation of a possible role of genetics in the development and expression of IgAN initially included linkage studies of multiplex pedigrees. Three loci on separate chromosomes have been identified, although the genes responsible for the linkage have not been defined (42, 47). Later, genome-wide association studies (GWAS), first in the United Kingdom and later with cohorts of patients and controls of European and east Asian ancestry, identified multiple loci associated with IgAN (now at least 30) (45, 48). The associated loci include a wide variety of genes, including some in the major histocompatibility complex involved in antigen processing and presentation. Other associated loci encompass genes involved in chemokine and B and T cell receptor signaling, regulation of the alternative complement pathway, genes encoding anti-microbial peptides α-defensins, and genes affecting NF-κB signaling, T cell–independent IgA class-switching, IgA plasma cell activation, IgA Fc receptor, and the *O*-glycosylation pathway (47, 49). Interestingly, analysis of 85 world populations performed by Kiryluk et al. (47) showed that a genetic risk score, based on 15 single-nucleotide polymorphisms, increased with progressive eastward and northward distance from Africa. Additional studies found a highly significant association between a genetic risk score and age at diagnosis; a greater genetic burden promoted an earlier onset of disease (46).

An alternative explanation of immunopathogenesis of IgAN based on the epidemiology and impact of Epstein-Barr virus (EBV) infection on the IgA system was recently proposed to elucidate the possible pathways in the geographic and race-associated differences in the prevalence of IgAN (50). The structural characteristics of IgA in CIC and mesangial deposits and phenotypic profiles of circulating IgA-secreting cells from IgAN patients revealed a remarkable concordance with results observed with *in vitro* EBV-infected B cells (18, 50–56), including the predominant secretion of pIgA1 with poorly galactosylated glycan chains (**Table 1**). Therefore, we initiated studies of phenotypes of EBV-infected B cells from the peripheral blood of IgAN patients and African American and White controls (50) to address a potential role of EBV in the pathogenesis of IgAN. These cells were analyzed with respect to the maturation profiles and the expression of cell-surface homing markers, including those involved in the characteristic lymphoid tissue distribution, and the ability to produce pIgA1 with poorly galactosylated glycans (50). The EBV-infected IgA^+^ B cells from IgAN patients displayed phenotypic characteristics very similar to those of IgA^+^ B cells infected *in vitro* with EBV (50–56).

**Table 1:** Concordance of properties of IgA produced by EBV-infected IgA-secreting plasma cells and IgA in circulating immune complexes and mesangial deposits.

	EBV-infected cells	CIC	Mesangial deposits
IgA Subclass	Only IgA1 secreted	IgA1	IgA1
Molecular forms of IgA	Polymer	Polymer	Polymer
Presence of J chain	+	+	+
Gd-IgA1	+	+	+
Lambda light chains	+	ND	+

CIC, circulating immune complexes; EBV, Epstein-Barr virus, Gd-IgA1, galactose-deficient IgA1; J, joining; ND, not determined; + states for "yes".

Based on published data (2, 3, 8–21, 50–56)

## 2 Evidence for the autoimmune nature of IgAN

**Figure 1:**
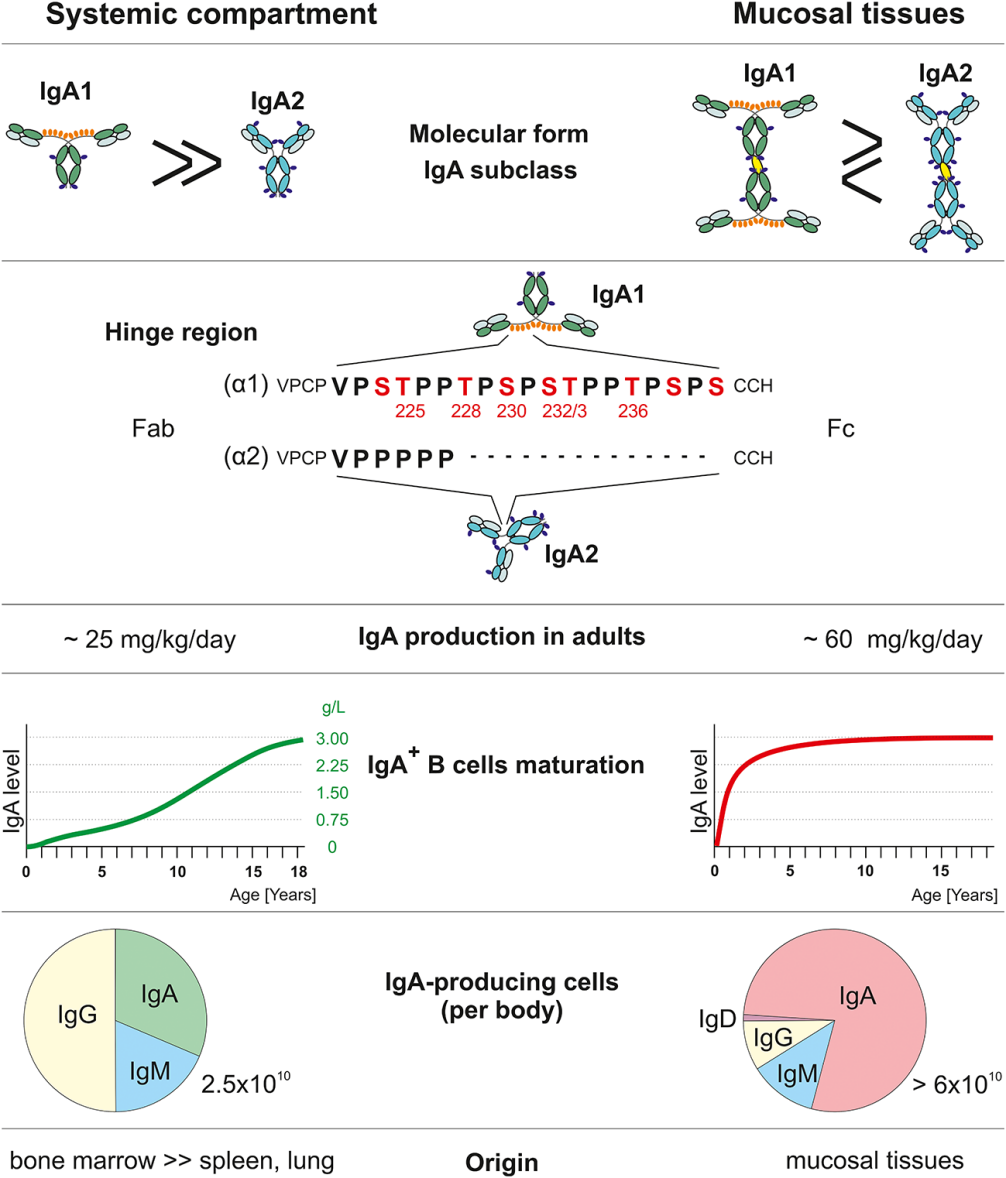
Comparison of systemic and mucosal IgA compartments. Systemic and mucosal compartments differ in proportions of the IgA subclasses, amount of IgA produced daily, dynamics of IgA production relative to normal adult values, the proportion of individual Ig isotype-positive cells, and tissues with IgA-secreting plasma cells. IgA1 heavy chain (α1) has, in contrast to IgA2 (α2), a unique hinge region with an additional 13 amino acids that include Thr and Ser residues which may be glycosylated. Red-highlighted amino acids may be *O*-glycosylated (55, 62–70).

**Figure 2:**
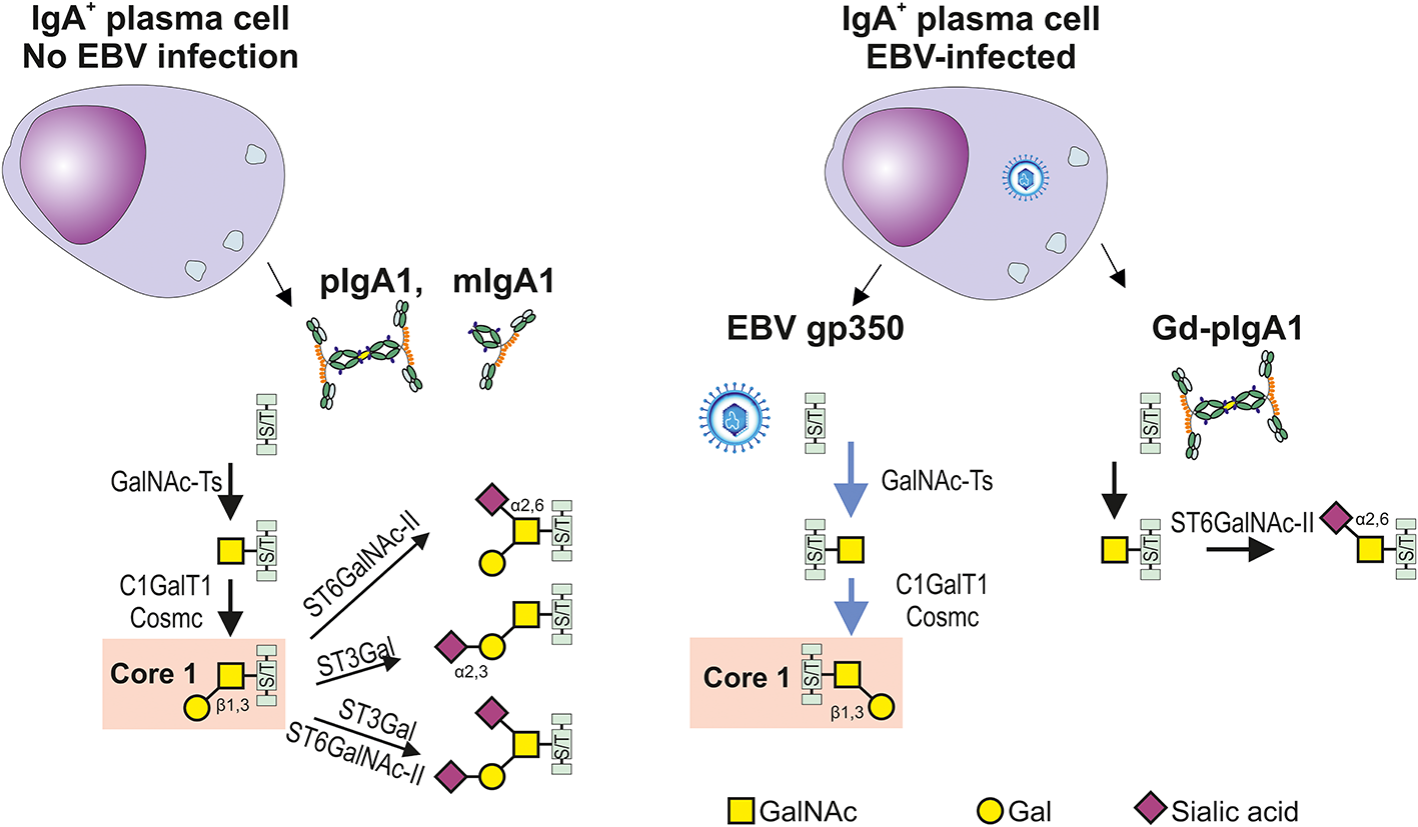
Differences in glycosylation pathways in EBV-infected or non-infected IgA1-producing cells. In the healthy conditions, IgA1-producing plasma cells generate IgA1 with hinge-region *O*-glycans; the prevailing form consists of the *N*-acetylgalactosamine (GalNAc) with β1,3-linked galactose (Gal) forming the Core 1 structure (also called T antigen) and its mono- and di-sialylated forms. *O*-glycosylation is a highly complex process involving about 50 glycosyltransferases and occurs in the Golgi complex. *O*-glycosylation is initiated by one of several *N*-acetylgalactosaminyltransferases (GalNAc-Ts) which catalyze the transfer of GalNAc to the Ser or Thr residues (S/T), leading to formation of Tn antigen. GalNAc-T2 is probably an essential enzyme responsible for galactosylation of IgA1; however, other GalNAc transferases are also expressed in B cells and could participate in this process (64, 65, 71–73). Formation of Tn antigen is followed by the addition of Gal catalyzed by only one known Core1 β1,3-galactosyltransferase 1 (C1GalT1) and its chaperon Cosmc. Core 1 can be expanded with sialic acid(s) attached, by several sialyltransferases to Gal, GalNAc, or both. The process is catalyzed by Galβ1,3GalNAc α2,3-sialyltransferase (ST3Gal) (72) or/and a α2,6-sialyltransferase (ST6GalNAc-I or ST6GalNAc-II), respectively (74). Replicating EBV-infected IgA1^+^ cells can produce EBV gp350 and IgA1. C1GalT1 participates in the parallel formation of Core1 on gp350 and IgA1, leading potentially to a relative C1GalT1 deficiency and generation of *O*-glycans with terminal GalNAc with or without α2,6 attached sialic acid. Preterminal sialylation of Tn antigen increases formation of Gd-IgA1 (75). .

Studies of the composition of CIC and mesangial deposits revealed that they consist of IgA exclusively of the IgA1 subclass, IgG, C3 of the complement cascade, and sometimes IgM (2, 3, 10, 11). These findings prompted the search for the participating exogenous or possibly endogenous antigens involved in CIC formation. Although antigens of the microbial and food origin have been sought as components of CIC or immune complexes in the mesangium, no uniformly prevalent antigen was identified (57–60). The possible autoimmune character of IgAN was postulated because of the exclusive presence of the IgA1 subclass in complexes with IgG,C3, and soluble IgA Fc receptor (FcαRI or CD89) in the absence of other identifiable components (10). Recently it was reported that CD89 represents critical factor for mesangial proliferation in childhood IgAN (61). As discussed below, human and hominoid-primate IgA1 has, in contrast to IgA2, a unique HR of the α1 heavy chains characterized by an additional 13 amino acids that include Thr and Ser residues which may be glycosylated (**Figure 1**) (62). Comparative evolutionary studies of the Ig HR clearly indicate a recent insertion of a gene segment encoding for the HR of IgA1 into phylogenetically older IgA2 (62). Furthermore, IgA1 from IgAN patients displayed an aberrant glycosylation pattern with the characteristic galactose (Gal) deficiency in the *O*-linked glycans in the HR (**Figures 1**, **2**) as revealed by reactivities with relevant lectins or monoclonal antibodies specific for Gd-IgA1, or direct biochemical analyses of glycans of IgA1 eluted from glomerular immune complexes (2, 3, 7, 8, 12, 14, 18, 71, 76). Gd-IgA1 in CIC and in mesangial deposits is in the polymeric form, as demonstrated by the elution profiles of IgA from dissociated CIC, and reactivity of mesangial IgA1 with secretory component (SC) – the extracellular part of the polymeric IgA receptor (pIgR) expressed on epithelial cells – which binds exclusively pIgA and IgM with J chain (2, 21, 77–80).

Gd-pIgA1 is recognized by antibodies specific for terminal N-acetylgalactosamine (GalNAc) residues in the *O*-linked glycans in the HR of Gd-pIgA1 which may contribute to the conformational alteration of HR and generation of new antigenic determinants (2, 3, 21, 23, 77, 81–83). It is thus evident that the structurally unique HR of human IgA1 in IgAN patients (**Figure 1**) resulted in the appearance of novel antigenic determinants. These epitopes are recognized by ubiquitous, naturally occurring, antibodies (21, 23, 84, 85) or those that probably evolve due to the affinity maturation (86), ultimately leading to formation of nephritogenic CIC that deposit in the glomerular mesangium (2, 3). The universal presence of IgG in mesangial deposits was demonstrated using anti-IgG nanobodies which selectively recognize potentially hidden antigenic determinants of IgG in mesangial immune complexes of IgAN patients (87). Furthermore, somatic mutations in the variable regions of heavy chains may modulate the affinity of autoantibodies against Gd-IgA1 (86). The presence of naturally occurring antibodies in sera of all healthy individuals, specific for the HR-containing Fab fragment of some IgA1 myeloma proteins, suggested that these IgA1 proteins exhibit unique antigenic determinants not present in IgA2 myeloma proteins (23, 86, 87). Importantly, parallel determination of such IgA1-specific antibodies revealed significantly increased levels in sera of IgAN patients (21, 77, 85). There are several sites in the HR of the IgA1 heavy chains that display a high degree of heterogeneity in their *O*-linked GalNAc without Gal (63, 64). The specific site(s) with the *O*-linked GalNAc that serves as the epitope(s) recognized by autoantibodies to form CIC that accumulate in the mesangium of IgAN patients has not been identified (65). Based on the reactivities of IgG anti-Gd-IgA1 antibodies with the HR fragments generated by the proteolytic cleavage of IgA1 myeloma protein, we propose that GalNAc on Thr228 and Thr233 may be involved (66).

However, the basis for the induction of these naturally occurring GalNAc-specific antibodies has not been conclusively established. Certain microorganisms, including EBV, express *O*-linked glycan chains on their surfaces which may induce such antibodies (88–93). Gp350/220 EBV complex is expressed on the virion surface at high density, thereby allowing efficient cross-linking of the CD21 and activating resting B cells to proliferate. Gp350 is heavily glycosylated with *N*- and *O*-linked glycans, together contributing 60% of its total molecular weight (88, 89).

The biochemical and biological activities of immune complexes containing Gd-pIgA1 and IgG were first reported after their characterization and isolation from sera of IgAN patients (3, 10, 21, 25, 77). The specificity of antibodies for GalNAc residues in *O*-linked glycans of the IgA1 HR was demonstrated by the inhibition of re-association of acid-dissociated immune complexes in the presence of other GalNAc-containing glycoproteins or free GalNAc (21). The biological properties manifested as proliferation of mesangial cells induced *in vitro* were dependent on the molecular mass of such complexes. Those of molecular mass ~700-1,000 kDa displayed the stimulatory effect whereas smaller complexes did not (3, 25, 26). Thus, we concluded that immune complexes composed of Gd-pIgA1 and GalNAc-specific IgG in the circulation of IgAN patients are nephritogenic and responsible for the clinical expression of IgAN (1–3, 6).

## 3 Uniqueness of human IgA system

### 3.1 Structural features relevant to IgAN

The fact that in humans IgA is produced in quantities that twice exceed the combined production of IgG and IgM (IgA ~70 mg/kg/day; IgG ~25 mg/kg/day; IgM ~7 mg/kg/day) (67) is infrequently acknowledged in the current literature. In contrast to other Ig isotypes, IgA occurs in several molecular forms: in plasma, ~95% of IgA is present as monomers (m) and ~ 1-5% as dimers and tetramers (62, 67). In external secretions, dimeric and tetrameric secretory IgA (S-IgA) with ~60% dimers and ~40% tetramers are dominant (62, 67, 94). With the exception of hominoid primates, serum IgA in other animal species is present mostly in dimeric form (95). In humans and hominoid primates, IgA occurs in two subclasses, IgA1 and IgA2; in other species (62, 95, 96), with the exception of lagomorphs, there is only a single IgA isotype, structurally similar to human IgA2 (62, 95). Thus, in comparison to phylogenetically older Ig isotypes, IgM analogs, IgG, and IgA2, it is apparent that IgA1 is phylogenetically a recent Ig isotype (95). The most obvious structural difference between IgA1 and IgA2 is in the unusual HR of IgA1 (**Figure 1**). The origin of the gene segment encoding the HR of IgA1 remains enigmatic. Most interestingly, this segment of IgA1 is the only known substrate for the family of structurally highly diverse proteases of bacterial origin capable of the cleavage of IgA1 into the Fab and Fc fragments (97). Other differences between the α1 and α2 heavy chains include allotypic determinants associated with the IgA2 isotype (62). Furthermore, antibodies specific for a variety of antigens differ in their association with the IgA1 and IgA subclasses (98).

IgA1 and IgA2 also differ in their glycosylation patterns (62, 63, 99). IgA1 contains *O*-linked oligosaccharide chains in the HR that are absent from Igs of all other isotypes except IgD (100). In the IgA1 HR, there are nine Thr and Ser residues to which GalNAc may be attached (62, 63). Analyses of *O*-linked glycans in monoclonal or polyclonal IgA proteins revealed a significant variability (63).

Based on our recent analyses of light chains associated with surface (s)IgA^+^ B cells, the dominant expression of λ chains was observed (101). Interestingly, the dominance of λ chains in mesangial deposits and pIgA1 in the circulation of patients with IgAN has been reported in many studies (102–108). Importantly for the elucidation of the IgA1 binding to mesangial cells, it appears that IgA1λ displays marked charge differences leading to enhanced binding (108). Because Gd-pIgA1 is present in the high-molecular-mass form of CIC and anti-Gd-IgA1 is detectable in a free form in sera of IgAN patients, it is obvious that CIC were generated in the antibody-excess zone. The biological effects of CIC and efficiency of their removal from the circulation are related to their molecular mass. Monomeric and polymeric IgA in their free forms display relatively short half-lives in the circulation (~4-6 days) and are effectively catabolized by hepatocytes which on their surfaces express the asialoglycoprotein receptor specific for Gal and GalNAc residues of glycoproteins, including IgA (68, 109, 110). However, the IgA1-containing CIC of the high molecular mass in sera of IgAN patients do not reach the space of Disse in the liver. Thus, it is likely that, due to the larger size of glomerular endothelial fenestrae, they enter the mesangium where they induce stimulation and proliferation of mesangial cells (2, 3).

### 3.2 Cellular aspects of IgA production

Plasma cells producing polymeric or monomeric IgA1 or IgA2 display a characteristic tissue distribution (62, 67, 69, 70, 111). Circulatory mIgA1 is produced by mainly plasma cells in the bone marrow; smaller amounts are secreted by plasma cells in systemic lymph nodes and spleen (62, 67, 111). Plasma cells in mucosal tissues produce pIgA but the tissue distribution of IgA1- or IgA2-producing cells displays a characteristic pattern: in the respiratory and upper alimentary tracts, IgA1-producing cells are present in higher numbers than are IgA2-producing cells, while IgA2-producing cells are dominant in the large intestine (62, 69, 70, 111). In the bone marrow, ~40-50% of plasma cells produce IgA; in contrast, in the intestines, ~90% of plasma cells produce IgA (55, 62, 69, 70, 109, 111) (**Figure 1**). This distribution of cells producing IgA1 or IgA2 in monomeric or polymeric forms is in agreement with quantitative data concerning the production and metabolism of IgA (109).

It is assumed that expression of Igs on surfaces of B cells precedes production of Igs of the same isotype after their differentiation into Ig-secreting plasma cells. In the case of sIgA^+^ B cells in peripheral blood, widely variable numbers of sIgA^+^ cells have been reported, partially due to the differences in the reagents and methods used to identify such cells. Importantly for the differentiation of cells ultimately secreting IgA1 or IgA2, the earlier expression of sIgA is not necessary; sIgM^+^ B cells may also directly differentiate into the IgA–secreting cells without prior expression of sIgA (112). This point is of considerable importance in the explanation of seemingly discrepant data concerning the phenotypes of B cells, including expression of sIg isotype and their history of EBV infection. The total numbers of IgA-producing cells in mucosal and systemic tissues greatly exceeds the numbers of IgG- and IgM-producing cells (**Figure 1**), thus explaining the pronounced dominance of IgA production over that of other Ig isotypes in humans (62, 67). Interestingly, recent data indicate that IgA-producing cells in the intestine exhibit a life-span of 10-20 years that is enormously extended compared with that of IgG- or IgM-producing cells (113). This surprising finding has a great impact of the physiology of the entire IgA system. Currently, it is not known whether the EBV-infected Gd-pIgA1λ-producing cells in other mucosal tissues and the bone marrow display such remarkable longevity.

### 3.3 Independence and different maturation of systemic and mucosal IgA cells

In addition to differences in molecular forms and tissue distribution of cells producing IgA, the systemic and mucosal compartments also exhibit remarkable degree of independence and maturation patterns (**Table 2**). IgA produced as monomers in the bone marrow and other systemic lymphoid tissues remains almost entirely in the circulation with a 4-5 day half-life and is catabolized in the liver; importantly, only trace amounts appear in external secretions (62, 67, 68, 109, 110). In contrast, pIgA produced in mucosal tissues is selectively transported by a receptor-mediated mechanism into external secretions (114). This receptor is specific for pIgA and IgM containing J chain and is expressed on mucosal epithelial cells of the intestinal, respiratory, and genital tracts and ductal cells of mucosa-associated glands (salivary, lacrimal, genital, and lactating mammary glands) (114). From the quantitative point of view, the amount of IgA produced in mucosal tissues greatly exceeds the amount of IgA generated systemically in the bone marrow (**Table 2**, **Figure 1**).

**Table 2:** Differences and independence of the systemic and mucosal compartments of the IgA system.

	Systemic	Mucosal
Quantities of IgA produced per kg body weight/day	~ 25 mg	~ 50 mg
Fate of IgA	Half-life 4-5 days Catabolized in liver	Transported into external secretions
Site of production	Bone marrow >> spleen > lymph nodes	Mucosal tissues
Number of IgA-producing cells	2.5 x 10^10^	~6 x 10^10^
Maturation	Adult levels reached in adolescence	Adult levels reached in 2-3 years, highly variable
IgA: molecular forms	95-99% mIgA	~95% pIgA
		Dimers (~60%) Tetramers (~40%) Contains J chain
Subclasses	IgA1 ~ 85% IgA2 ~ 15%	Variable Upper respiratory IgA1 > IgA2 Large intestine IgA2 > IgA1

m, monomeric; p, polymeric

Based on published data (51, 55, 67, 68, 92, 103, 113–123)

In general, serum IgA displays a naturally highly delayed maturation pattern, manifested as absent or trace amounts of IgA in cord blood with a strongly age-dependent increase in levels in the circulation; adult serum levels of IgA are reached during adolescence (**Figure 1**) (115–120, 124, 125). In contrast, adult levels of secretory IgA (S-IgA) are attained at ~ 1-2 years of age. Thus, there is an extended period of physiologically normal IgA deficiency in the systemic compartment. These serological data are corroborated by immunohistochemical studies of IgA-producing cells in systemic and mucosal lymphoid tissues demonstrating an age-dependent paucity of such cells in lymphoid tissues (70, 121–123, 126, 127). This physiological delay in maturation of the systemic IgA compartment is of enormous importance in the immunopathogenesis of IgAN when related to the racial differences in EBV infection (50).

**Figure 3:**
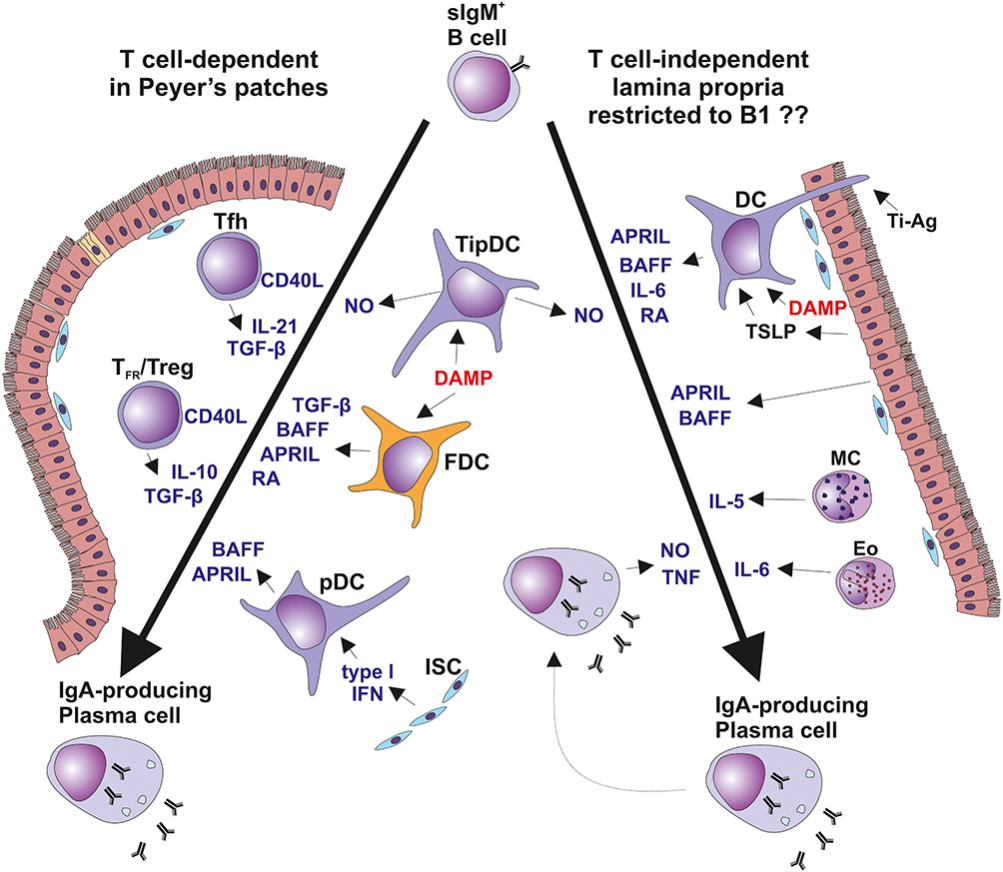
T cell-dependent and T cell-independent IgA isotype switching. Surface IgM-positive (sIgM^+^) B cells are induced to undergo isotype switch by i) T cell-dependent manner in Peyer’s patches and other mucosa-associated lymphoid follicles (left panel) or by ii) T cell-independent manner in the vicinity of mucosal epithelial surfaces in diffuse lymphoid tissues of lamina propria mucosae (right panel) (128–134). In Peyer’s patches, B cells differentiation depends only partially on the cognate help signals from Tfh exposing CD40L and secreting TGF-β and IL-21. In concert, FDCs secrete BAFF, APRIL, RA, and TGF-β in response to DAMP stimulation. Furthermore, FDCs present native antigens to B cells to support crosslinking by BCR. Peyer’s patches contain also a TipDCs which render B cells more sensitive to TGF-β due to NO-induced enhancement in the expression of TGF-β receptor. Peyer’s patches contain also pDC secreting BAFF and APRIL upon stimulation by type I interferon from the ISC (128, 134). Diffuse lymphoid tissues of the intestinal lamina propria contribute mostly to the T cell-independent Ig switching in B1 cell subset. Besides several subsets of MC and DC, local PCs are involved in this process. TipDC are typical for lamina propria and they act similarly to their counterpart in Peyer’s patches. In addition, DC expressing TLR5 member of the DAMP family are stimulated by flagellin to secrete RA and IL-6. DAMP-activated DC co-stimulated by TSLP from epithelial cell produce BAFF and APRIL. Some DC extend their dendrites through the epithelial cell junction or across the M cells into intestinal lumen to sample antigens for recycling and presentation in unprocessed form to B cells, as the T cell-independent antigens. PC maturation and survival could be supported by mast cells producing IL-4, IL-5, IL-6, and BAFF. Furthermore, Eo could contribute to PC survival by secretion of IL-6. Finally, local IgA-secreting PC were identified as a producers of TNF and iNOS (128, 129, 135, 136). APRIL, a proliferation-inducing ligand; BAFF, B cell activating factor; BCR, B cell receptor; DAMP, danger-associated molecular pattern; DC, dendritic cells; Eo, eosinophils; FDC, follicular dendritic cells; iNOS, TNF-inducible nitric oxide synthase; ISC, intestinal stromal cells; MC, mast cells; NO, nitric oxide; PC, plasma cells; pDC, plasmacytoid dendritic cells; RA, retinoic acid; Tfh, follicular T helper cells; T_FR,_ follicular regulatory T cells; TipDC, TNF- and iNOS-producing DC; TSLP, thymic stromal lymphopoietin.

**Figure 4:**
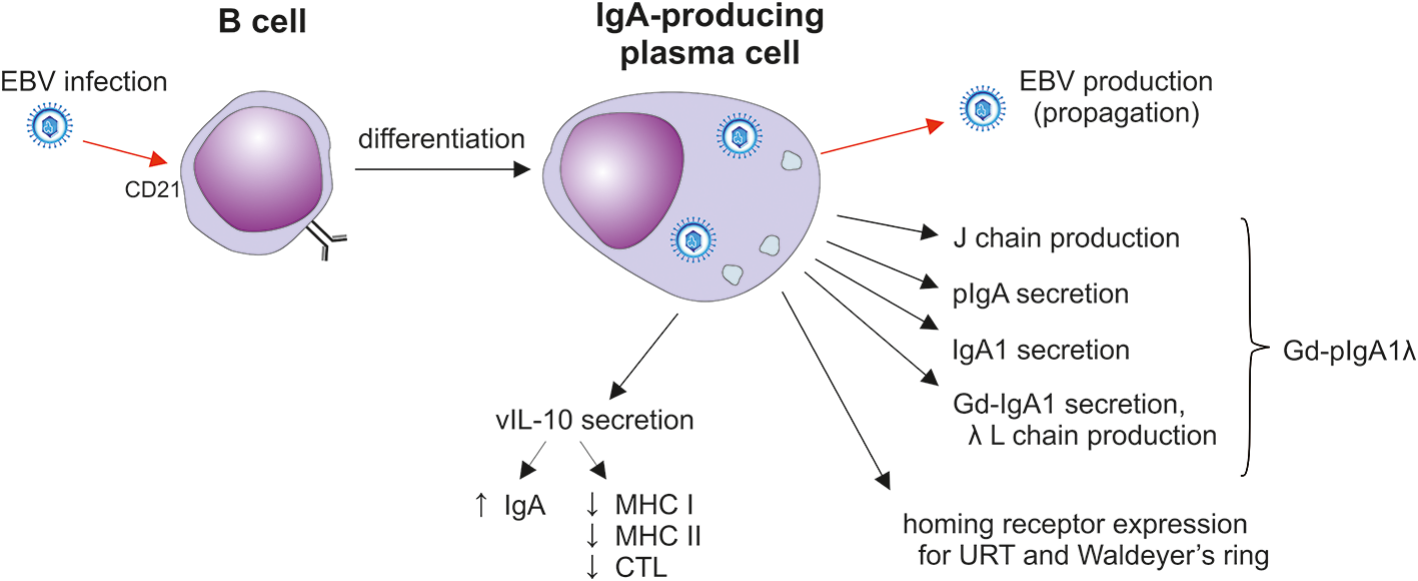
The impact of EBV infection on sIgA1^+^ B cells. After the initial mucosal infection, the virus remains in resident memory B cells; upon activation, Igs and EBV are produced in plasma cells (141–143). EBV-infected plasma cells secrete J-chain-containing Gd-pIgA1 with preferentially λ light chains. Such cells also display homing receptors involved in the selective population of the upper respiratory mucosa. In addition, vIL-10 is likely to support the differentiation of cells into IgA producers and probably suppresses the cytotoxic activity of CTLs (141, 143, 144). CTL, cytotoxic T lymphocytes; Gd-IgA1, galactose-deficient IgA1; J, joining; L light; URT, upper respiratory tract.

The differentiation of B cells into IgA-secreting plasma cells proceeds in T cell-dependent or -independent pathways regulated by substances involved in Ig-isotype switching (**Figure 3**) (128, 135). The progression of sIgM^+^/D^+^ B cells into IgA-producing plasma cells is regulated by products of T cells as well as cells of the non-T cell phenotype which mediate the sequential steps involving Ig isotype switching, proliferation, and terminal differentiation (**Figure 3**) (128, 129, 135, 136). Particularly, TGF-β and IL-10 participate in Ig isotype switching and IL-10 increases terminal differentiation into IgA-producing plasma cells. Both chemokines are secreted by T cells and epithelial cells (129, 136). Thus, IL-10 may enhance production of IgA to reach high circulating levels, a characteristic feature of IgAN (1–3, 137–139). One explanation of these high levels is the activity of the EBV lytic gene BCRF1; that encodes a homolog of cellular IL-10, designated as vIL-10, that accelerates terminal differentiation of B cells into IgA-producing plasma cells (129, 136, 140). vIL-10 also has immunosuppressive activity. vIL-10 is analogous to cellular IL-10 in its suppression of INF-γ synthesis in human peripheral blood mononuclear cells and reduction of responses of NK and cytotoxic T cells (**Figure 4**) (145, 146).

## 4 The impact of EBV infection on B cell differentiation and IgA production

The pathways of differentiation of lymphocytes of B cell lineage into IgA-producing cells have been extensively studied using polyclonal stimulation with various cytokines, pokeweed mitogen (PWM), and EBV (51–55, 135, 147–151). EBV infects B cells in the earliest stages of their differentiation pathway (**Figure 4**). Human pro-, pre-, immature, and mature B cells are EBV infectable due to the presence of an EBV receptor, CD21 (52). Interestingly, as EBV-infected B cells mature, the transition of pro– and pre–B cells into phenotypically characterized plasma cells is not accompanied by a parallel synthesis of Igs (52). This lack of production is due to the “sterile” differentiation pattern, with the failure of VDJ rearrangement (52). Such plasma cells contain abundantly expressed J chain that is otherwise involved in the polymerization of IgA and IgM (62). In cell culture, J chain is not secreted in the free form into the supernatants and remains strictly in the intracellular compartment (52). EBV infection of peripheral blood B cells induces their differentiation into lymphoblasts, plasmablasts, and plasma cells secreting Ig of all major isotypes (51). Although EBV induces intracellular production of IgA of both subclasses, only IgA1 is secreted into cell culture supernatants after extended incubation (53, 54). This finding is of considerable importance in IgAN because the IgA in elevated levels in patients’ sera is exclusively of the IgA1 subclass (1–3, 9). Furthermore, most of the secreted IgA1 is in the polymeric, J chain-containing, form (51, 55) and is Gal-deficient (**Table 2**) (18).

The preferential synthesis of IgA1 that is Gal-deficient may be due to competition between IgA1 and the gp350 protein of EBV for galactosylation. Galactosylation of GalNAc residues in the HR of IgA1 involves the enzymatically-mediated transfer of Gal from the donor, UPD-Gal, to the recipient GalNAc residues (64). Importantly, the galactosylation of the IgA1 HR in EBV-infected plasma cells proceeds with the parallel production of EBV with its heavily *O*-glycosylated gp350 glycoprotein (67, 88, 89). Thus, it is conceivable that the GalNAc residues on the HR of IgA1 and gp350 of EBV compete for UDP-Gal as well as access to the requisite enzymes C1GalT1 and Cosmc, resulting in the reduced galactosylation of IgA1 HR (**Figure 2**).

In EBV-infected cells, the activity and gene expression of several enzymes involved in the synthesis of the *O*-glycans in the IgA1 HR are altered (18). The activity of **β**1,3-galactosyltransferase that adds Gal to GalNAc is deceased. The galactosylation is further stressed by reduced expression of encoding gene, C1GalT1, and the gene encoding Cosmc, the chaperone for **β**1,3-galactosyltransferase that maintains its enzymatic activity. Furthermore, another enzyme, ST6GalNAc, exhibits increased activity and its gene is overexpressed. The resulting enhanced **α**2,6-sialylation prevents attachment of Gal to GalNAc-S/T in the IgA1 HR, thereby accentuating synthesis of Gd-IgA1 (75).

Recently, Dotz et al. (152) found that decreased sialylation of IgA1 is associated with decreased estimated glomerular filtration rate (eGFR) in patients with IgAN. Although this study used mass spectrometry to analyze serum total IgA containing IgA1 and IgA2 in monomeric and polymeric forms, the finding may provide a new biomarker for monitoring disease activity. Two earlier reports confirmed that Gd-IgA1-specific autoantibodies in IgAN patients bound more Gd-IgA1 after removal of sialic acid (23, 86). Based on the observation of Dotz et al, acute removal of sialic acid from Gd-IgA1 HR, for example due to infection by neuraminidase-secreting viruses (such as influenza) or bacteria (such as pneumococci), could contribute to increased amounts of Gd-IgA1 in the circulation, leading to enhanced binding of autoantibodies and formation nephritogenic CIC. This scenario may explain the clinical association of macroscopic hematuria with mucosal infection in patients with IgAN (3). Because mucosal infection induces a general inflammatory response that includes stimulation of IgA-secreting cells and Gd-IgA1 production and because desialylation of IgA is the natural catabolism of IgA, the postulated contribution of infection-mediated desialylation of Gd-IgA1 to disease activity in IgAN should be tested in future studies.

EBV profoundly influences the expression of receptors on infected B cells, with the preferential expression of those involved their homing to tonsils and the upper respiratory tract (50, 153, 154). Thus, EBV, as well as other viruses (155), direct the ultimate tissue distribution of these cells through expression of pertinent homing receptors. In the case of EBV infection, integrin α4β7 (LPAM-1) is induced in the tonsils of patients with infectious mononucleosis, thereby allowing B cells to home to the gastrointestinal mucosa-associated lymphoid tissue (GALT) (156).

## 5 EBV infection

**Figure 5:**
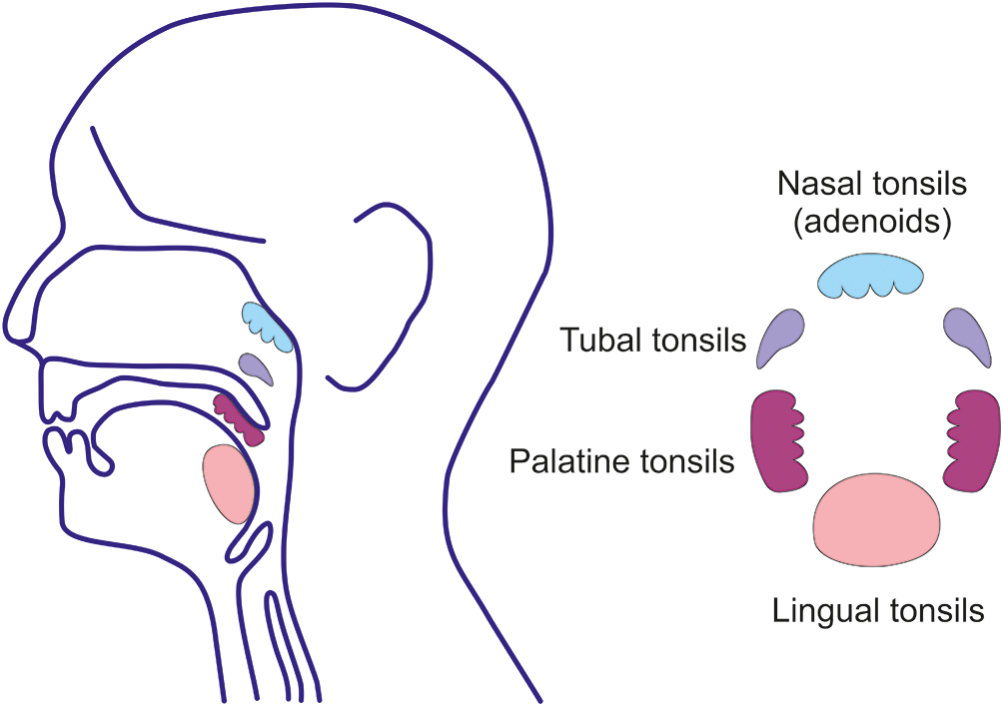
Waldeyer’s ring. Waldeyer’s ring is comprised of the nasopharyngeal tonsils (adenoids) attached to the roof of the pharynx, the tubal tonsils (adenoids) located at the pharyngeal aperture of the Eustachian tubes, the palatine tonsils in the oropharynx, and the lingual tonsils on the posterior third of the tongue. Tonsils are lymphoreticular and lymphoepithelial organs. Tonsillar epithelium invaginates and lines the tonsillar crypts enhancing the surface for direct contact with exogenous antigens to a surface of 350 cm^2^, predominantly in the palatine tonsils (162).

EBV as well as other herpesviruses establish life-long and latent residence in target cells of the host and evade elimination (141, 142, 157–160). EBV infects only humans (157, 160). The ensuing clinical manifestations depend on the type and magnitude of the induced immune responses and age of the host (141, 157–160). Acute EBV infection of children usually remains clinically silent (141, 158–161). EBV is present in saliva to provide an easy means to spread the virus to uninfected individuals. EBV crosses the epithelial barrier of the oral cavity and nasopharynx to infect susceptible B cells to induce their proliferation and maturation to the Ig-secreting plasma cells or to establish persistent residence (141, 142, 157). The most important lymphoepithelial tissue susceptible to EBV infection is Waldeyer’s ring which includes the adenoids and palatine, tubal, and lingual tonsils with adjacent draining lymph nodes (**Figure 5**) (141, 142, 157). EBV establishes persistent infection in resident, long-lived, memory B cells (142) and retains the ability to replicate in activated and differentiated plasma cells that produce antibodies of various isotypes and also release the virus (**Figure 4**) (141, 143, 144). In these oropharyngeal lymphoid tissues, the virus can initiate a new round of B cell and perhaps epithelial cell infection that leads to further shedding into the saliva (141). However, the possibility of EBV replication in epithelial cells remains controversial; virus in complexes with antibodies may be also internalized through the Ig receptors expressed on epithelial cells (141). The frequency of EBV-infected B cells is highly variable, ranging from 5 to 3,000 infected cells/10^7^ memory B cells in Waldeyer’s ring and peripheral blood; other lymphoid tissues (spleen and mesenteric lymph nodes) contain at least a 20-fold fewer infected cells (141, 142, 157). The virus replicates in terminally differentiated B cells (plasma cells) in Waldeyer’s ring but only a few cells participate in viral production (143, 157). However, the life-span of such infected plasma cells in the upper respiratory tract and oral cavity has not been determined. In mucosal tissues and the bone marrow, IgA-producing plasma cells persist for a surprisingly long time, up to 10-20 years (113). Asymptomatic EBV reactivation in oral mucosa-associated lymphoid tissues occurs periodically in most healthy subjects (94). This event may be caused by reactivation of EBV-infected resting memory B cells upon their entry into lymphoid tissue and physiologic stimulation through the B cell receptor, leading to terminal differentiation into plasma cells and activation of the viral replicative cycle, associated with expression of viral glycoprotein gp350.

Due to the presence of pIgA-producing cells with J chain in the bone marrow of IgAN patients, the glomerular IgA may originate in the bone marrow as well as mucosal tissues (163–165). However, the altered glycosylation pattern and the possible presence of EBV in the bone marrow of IgAN patients has not been addressed. It is conceivable that in these patients there are indeed Gd-pIgA1λ-producing cells in the bone marrow in addition to the lymphoid tissue in the upper respiratory tract and, perhaps, other mucosal tissues. In these tissues, EBV remains associated with infected memory B cells. Upon the stimulation of these cells, probably by infection with other microorganisms, some differentiate into plasma cells that produce Gd-IgA1λ and release EBV.

### 5.1 Epidemiology of EBV infection

Although ~95% of adults worldwide are EBV-infected (141, 142, 157, 158, 160, 161), there are significant differences in age at primary infection and the incidence of EBV-associated diseases (166, 167). Children from the birth to ~ 6 months of age are protected against EBV infection by maternal IgG antibodies acquired by transplacental transfer (167). Most importantly, socio-economic status, irrespective of the country or continent, is of primary importance (160, 161, 168–171). The number of children in a family, sharing of rooms and utensils, hygienic conditions, level of family income, cultural practices (such as maternal pre-chewing of food) (141, 159, 161, 171–173), and breastfeeding with milk containing EBV (174–176) may impact the likelihood and timing of EBV infection. Up to ~90% of children in families with unfavorable socio-economic situations become infected within the first year of life (166–168). This finding may be a relevant factor for early EBV infection of some African American, African Black, and Aborigine children in Australia (166–168). Importantly, EBV infection at a young age is generally asymptomatic (159–161). Furthermore, epidemiological data indicate that early EBV infection induces protective humoral and cellular immune responses resulting in the significantly reduced incidence of infectious mononucleosis and possibly some autoimmune diseases (174). In sharp contrast, individuals of a higher socio-economic status more often are infected during adolescence and have an increased frequency of EBV-associated diseases (169). Recent studies have shown an association between a progressively older age at primary EBV infection with a higher incidence of infectious mononucleosis and other EBV-associated diseases (173). Based on the above-described consequences of EBV infection of B cells as related to the naturally delayed maturation of the IgA system, it is possible that socio-economic status, in addition to genetic factors, plays an important role in the incidence of IgAN. Description of familial incidence of EBV infection with different clinical outcomes in individual family members also may be relevant in IgAN. Many first-degree relatives of IgAN patients have high blood levels of Gd-pIgA1 without any clinical or laboratory evidence of kidney disease (46, 177). However, EBV serology has not been performed to assess a possible contribution of EBV infection to the development of IgAN. Nevertheless, in view of the strongly age-dependent clinical manifestations of EBV infection, it is possible that the variable outcome is due to quantitative differences in the blood levels of Gd-pIgA1 and corresponding autoantibody to generate CIC with possible nephritogenic potential.

### 5.2 EBV involvement in human diseases

Although more than ~95% of adults worldwide are infected with EBV, relatively few individuals display a broad spectrum of EBV-associated diseases of infectious, autoimmune, or malignant nature (141, 161, 178–185). Apparently additional immunologic, genetic, and environmental factors contribute to the development of EBV-associated diseases (158, 169, 180, 181, 183–185). Infectious mononucleosis is a disease most commonly acquired at 15-25 years of age in developed countries (159, 161, 174, 185). However, the disease is uncommon in African Blacks and 30x less common in African Americans than in Whites (160, 161). Clinical symptoms of infectious mononucleosis appear most frequently in adolescents/young adults living in areas with high hygienic and socio-economic conditions. These patterns are reminiscent of the incidence of IgAN with well documented racial differences (1, 28, 29, 32, 33, 37).

EBV-associated malignancies include Burkitt’s lymphoma (in association with malaria or HIV infection), Hodgins’s disease, nasopharyngeal carcinoma, gastric carcinoma, and, possibly, multiple myeloma (178, 180). EBV infection has been also associated with many diseases of the autoimmune nature, including systemic lupus erythematosus, multiple sclerosis, rheumatoid arthritis, inflammatory bowel disease, and possibly Sjögren’s syndrome and others (169, 181–183, 185).

### 5.3 Low incidence of IgAN in individuals with early EBV infection

Because almost all adults, irrespective of race, gender, socioeconomic status, and other variable environmental factors, become infected with EBV, the obvious question concerns the relatively low incidence of EBV-associated diseases, including IgAN, in racially diverse populations. We propose that, in the case of IgAN, the timing of EBV infection plays an essential role. In children infected prior to the strongly age-dependent maturation of the IgA system, EBV infects the precursors as well as mature B cells of non-IgA phenotypes and the ensuing humoral and cellular responses effectively protect against later infection of sIgA^+^ B cells (50). Early EBV infection is usually asymptomatic, probably due to the effective elimination of EBV-infected cells by CD8^+^ cytotoxic T lymphocytes which diminishes with advancing age (169, 185). This scenario is likely a common mechanism in the appearance of several autoimmune diseases as related to the age of the individual (169, 181, 183, 185). The presence of Gd-IgA1 in the circulation of asymptomatic relatives of IgAN patients (46, 177) as well as in mesangial deposits in individuals without clinically manifested kidney disease (186) suggests that not only the level of Gd-pIgA1 but also the antigenic determinants in the HR of Gd-IgA1, and the level and perhaps specificity of corresponding IgG autoantibodies that lead to marked differences in the serum levels of CIC and, most importantly, CIC molecular properties, especially with respect to the molecular mass, play important roles in disease expression (24). An analogous situation occurs in serum sickness, in which CIC of various molecular masses (based on the proportion of antigen to antibody) are effectively eliminated and only those of relevant molecular mass deposit in the mesangium (187). Based on this principle, additional studies to examine the capacity of non-cross-linking monovalent antibodies to Gd-pIgA1 to block formation of nephritogenic CIC should be explored.

### 5.4 Potential role of EBV infection in the geographic distribution of IgAN

IgAN is the most common form of glomerulonephritis in many countries in Europe, North America, and Australia, and east Asia (1, 28–30). In contrast, the disease is rare in Africa, many Asian and South American countries, in indigenous Australian Aborigines, and is uncommon in African Americans (28, 29, 31–40, 188). The rarity of IgAN in African Americans is remarkable in the light of the findings of a recent GWAS study that found African ancestry consistently associated with higher serum IgA levels and greater frequency of IgA-increasing alleles compared to other ancestries and that a high serum IgA level was correlated with IgAN (189). However, this study did not test for an association of ancestry with serum levels of Gd-IgA1, a small fraction of serum total IgA and the autoantigen for development of IgAN, or examine the potential influence of environmental factors.

Based on the above-described impact of EBV infection of human B cells with respect to the cell differentiation, production of Gd-pIgA1λ, and expression of homing receptors involved in populating Waldeyer’s ring and the upper respiratory tract, we compared the frequency of EBV-infected B cells and their expression of sIg isotypes and homing receptors in White IgAN patients and healthy adult White and African American controls (50). In the IgAN patients, EBV-infected B cells displayed dominantly sIgA while in the African American controls such cells were missing and only sIgM/sIgD-positive cells were present (50). Furthermore, EBV-infected B cells from White IgAN patients more frequently expressed the α4β1 homing receptor for the upper respiratory tract and Waldeyer’s ring (50). In concert with previous *in vitro* studies, we proposed that EBV is intimately associated in pathogenesis of IgAN (**Figure 2**, **5**) (50).

Comparative epidemiological studies in various countries have revealed that the marked temporal, racial, and geographic differences in the acquisition of EBV infection are strongly related to socioeconomic status (172, 173, 176). In addition to the above-described factors, child care in nurseries (190) or, in the adolescence, entry into a university lead to a significant increase in EBV seroconversion (170, 171). Early EBV infection also occurs in African Americans, African Blacks, and in Australian and New Guinea indigenous populations; all children ages 1-5 years were EBV-seropositive (168). Importantly, the incidence of IgAN in these populations is significantly lower than in the Australian non-indigenous White population (36). Thus, age-related studies of EBV seroconversion in countries with the low-frequency of IgAN (e.g., New Zealand, South Africa, Sudan, Bangladesh, India, Saudi Arabia, Peru, and others) would be important. Early EBV seropositivity is also accompanied by a significant decrease in the incidence of infectious mononucleosis. However, the predicted trend in the delay of EBV acquisition is likely to be followed by an increase in the incidence of infectious mononucleosis (173, 191) and possibly IgAN.

## 6 IgAN, IgA vasculitis with nephritis, and EBV

IgA vasculitis (formerly known as Henoch-Schönlein purpura) is the most common vasculitis in children, characterized by leukocytoclastic inflammation and IgA in the small blood vessels in the skin, joints, intestines, and (in a minority of patients) kidneys (192–195). IgAN and IgA vasculitis with nephritis share some common clinical, laboratory, and pathology features, including increased levels of Gd-IgA1 in the circulation and accumulation of Gd-IgA1 in glomeruli, suggesting a related immunopathogenesis (17, 43, 196–199). In contrast, IgA vasculitis patients without nephritis have normal circulating levels of Gd-IgA1 (196, 197). Furthermore, serum Gd-IgA1 levels are elevated in many first-degree relatives of pediatric patients with IgAN and IgA vasculitis with nephritis (43, 196). Based on the mechanisms involved in the glycosylation of HR of IgA1 (196, 200) and the familial epidemiology of EBV infection, it is plausible to speculate that EBV is also involved in the aberrant glycosylation of IgA1 in IgA vasculitis with nephritis. Indeed, several case reports support this possibility (201–203). The occurrence of IgA vasculitis with nephritis in children seropositive for acute EBV infection has suggested a role for the virus in the acute syndrome (201–203).

### 6.1 IgAN, tonsillectomy, and EBV

After the initial mucosal infection through the oral and upper respiratory tract, EBV establishes a latent and persistent residence in Waldeyer’s ring (**Figure 5**) (141, 142, 157, 204). The virus remains in the resident memory B cells and, upon activation, EBV is produced by plasma cells (141–143) in the free or epithelial cell-associated forms and appears in the saliva. The lymphoepithelial oropharyngeal tissues function as mucosal inductive as well as effector sites (205–207). These tissues contain Ig-producing cells, including those secreting IgA (205, 206, 208). Several studies have suggested that the tonsils and cells in the adjacent structures are the dominant source of IgA, including Gd-pIgA1 with J chain, which enters the circulation (209–216). Indeed, cultured Ig-producing cells from tonsils secreted Gd-pIgA1 into culture supernatants. Therefore, tonsillectomy has been promoted for treatment of IgAN in combination with corticosteroids in some studies (217–223). Nevertheless, tonsillectomy has remained controversial as a treatment option for IgAN due to the discrepant reports summarizing the results of tonsillectomy in various countries (222–225).

The palatine tonsils represent the largest but certainly not the only component of the Waldeyer’s ring (205–208). Adenoids, lingual and tubal tonsils, other associated small lymphoepithelial oropharyngeal structures, and draining lymph nodes with resident EBV-infected B cells and their descendants remain a potential continual source of Gd-pIgA1, albeit in lower amounts. Furthermore, other mucosal tissues may also contribute to the pool of Gd-pIgA1 in the circulation. EBV-infected B cells are found rarely in systemic lymphoid tissues, including the spleen and mesenteric lymph nodes (141), probably due to the lack of the relevant homing receptors. Nonetheless, a small portion of these cells also express gut-associated α4β7 homing receptors (50) and, upon differentiation, could contribute Gd-pIgA1λ to the circulatory pool. It is conceivable that the IgA1-producing plasma cells in the bone marrow in IgAN patients also produce Gd-pIgA1λ. This possibility remains to be explored because of the bone marrow has been considered to be the source of circulatory pIgA1 in IgAN patients (163, 164).

## 7 Conclusions

Based on the pleiotropic impact of EBV infection on B cells as related to the stage of maturation of the IgA system, the production of Gd-pIgA1λ as the autoantigen, and the preferential expression of homing receptors specific for the upper respiratory tract, we propose a novel pathway in the immunopathogenesis of IgAN. Although almost all adults are infected with EBV, there are marked differences in the time of infection among individuals of diverse racial and, more importantly, socio-economic backgrounds. Most African Americans, African Blacks, and Australian Aborigines are infected with EBV in very early childhood (1-2 years of age) without overt clinical symptoms. At that time, the IgA system is physiologically immunodeficient, manifested as absent or low serum levels of IgA and a paucity of IgA-producing cells in lymphoid tissues. Consequently, when very young children are exposed to EBV, the virus enters B cells of non-IgA isotype. The ensuing humoral and cellular immune responses prevent subsequent infection of IgA^+^ B cells during EBV reactivation at older ages when IgA B cells are more numerous. EBV infection of IgA-secreting cells markedly increases the fraction of synthesized IgA that is Gd-pIgA1λ. Therefore, EBV infection at a very young age may significantly reduce the lifetime risk of development of IgAN. Thus, the low incidence of IgAN in the above specified populations may reflect immunological, age-related, genetic, and pronounced socio-economic differences from populations with higher incidences of IgAN with respect to the frequency of early acquisition of EBV infection.

## Author contributions

JM conceived and designed the manuscript and wrote the original draft. All authors contributed to the article and approved the submitted version.
